# Yeast Two-Hybrid Screen Identifies PKA-Riα Interacting Proteins during Mouse Spermiogenesis

**DOI:** 10.3390/genes12121941

**Published:** 2021-11-30

**Authors:** Kunyu Shi, Lele Yang, Xueqing Zhuang, Lan Zhang, Huayu Qi

**Affiliations:** 1 GMU-GIBH Joint-School of Life Sciences, Guangzhou Medical University, Guangzhou 511436, China; shi_kunyu@gibh.ac.cn (K.S.); Zhuang_xueqing@gibh.ac.cn (X.Z.); 2 CAS Key Laboratory of Regenerative Biology, Guangzhou Institutes of Biomedicine and Health, Chinese Academy of Sciences, Guangzhou 510530, China; yang_lele@gibh.ac.cn; 3 Guangdong Provincial Key Laboratory of Stem Cell and Regenerative Medicine, Guangzhou Institutes of Biomedicine and Health, Chinese Academy of Sciences, Guangzhou 510530, China

**Keywords:** PKA, PKA-RIα, MENA, yeast two-hybrid screen, spermiogenesis

## Abstract

cAMP-dependent protein kinase (PKA) signaling plays various roles during mammalian spermatogenesis, ranging from the regulation of gene expression to the modulation of sperm motility. However, the molecular mechanisms that govern the multifaceted functions of PKA during spermatogenesis remain largely unclear. We previously found that PKA regulatory subunit I α (RIα) and catalytic subunit α (Cα) co-sediment with polyribosomal fractions of mouse testis lysate on sucrose gradient and the stimulation of PKA activity facilitates protein synthesis in post-meiotic elongating spermatids, indicating that type I PKA is intricately associated with protein translation machinery and regulates protein synthesis during mouse spermiogenesis. Since PKA activity is often regulated by interacting proteins that form complexes with its regulatory subunits, the identification of PKA-RIα interacting proteins in post-meiotic spermatogenic cells will facilitate our understanding of its regulatory roles in protein synthesis and spermiogenesis. In the present study, we applied a yeast two-hybrid screen to identify PKA-Riα-binding proteins using a cDNA library generated from mouse round and elongating spermatids. Numerous proteins were found to potentially interact with PKA-RIα, including proteostasis modulators, metabolic enzymes, cytoskeletal regulators, and mitochondrial proteins, many of which are specifically expressed in testes. Consistently, the examination of MENA (mouse ENA/VASP homolog) in developing mouse testes suggested that post-meiotic spermatogenic cells express a short isoform of MENA that interacts with PKA-RIα in yeast two-hybrid assay. The identification of PKA-RIα interacting proteins provides us solid basis to further explore how PKA signaling regulates protein synthesis and cellular morphogenesis during mouse spermatogenesis.

## 1. Introduction

Male gamete, spermatozoa, plays important roles during the propagation of genetic information, maintenance of animal species, and initiation of developmental programs following fertilization. Mammalian spermatogenesis, the developmental process during which sperm develop from spermatogonial stem cells, encompasses three consecutive stages: cell proliferation, meiosis, and cellular morphogenesis, the last of which gives rise to eventual spermatozoa with the unique cellular morphology that fits their biological functions [1,2]. Spermatogenic cells that reside within seminiferous tubules in male gonads are constantly intrigued by extracellular signals to undertake changes of cellular states. This is accompanied by the re-programming of gene expression, which can be regulated at both the transcriptional and post-transcriptional levels [3,4]. Protein mass spectrometry studies have suggested that mouse spermatozoa contain around 3–4 thousand proteins, of which both the quality and quantity appear to be important for sperm to function properly [5,6,7]. Genetics studies in various animal models, including *Drosophila*, *C. elegans*, and mice, have shown that the deletions or mutations occurring in sperm proteins are the direct causes of male sterility [8,9]. In human infertility diseases such as oligo-astheno-teratozoospermia (OAT), malformed spermatozoa are frequently observed, the etiology of which remains largely unclear [10,11,12,13]. Thus, understanding how spermatogenic cells regulate their proteome would facilitate not only unveiling molecular mechanisms of spermatogenesis but also the development of diagnostics and treatment of human infertility.

Spermatogenic cells enter cellular morphogenesis, also known as spermiogenesis, when meiosis is completed and cells become haploid. Research in the past has unveiled many important aspects of the molecular mechanisms that govern spermiogenesis, including key transcription factors that regulate post-meiotic gene expression, such as CREM (cAMP response element modulator) and TBPL1 (TATA Box-Binding Protein Like 1, also known as TLF/TRF2) [14,15]. However, due to the genome reprogramming and chromosomal condensation that occur shortly after spermiogenesis starts, gene transcription is soon turned off and hundreds of sperm proteins are synthesized using stored messenger RNAs [4,16], suggesting an inevitably important post-transcriptional regulation of spermatogenesis. How haploid spermatogenic cells (a.k.a. spermatids) initiate the cellular morphogenesis and what signals induce protein synthesis during spermiogenesis are not clear. In this regard, cAMP-dependent protein kinase A (PKA) is one of the signaling pathways that has been implicated in participating in the regulation of gene expression and the motility of mature sperm [17]. However, molecular mechanisms that underlie functional roles of PKA during spermiogenesis remain largely unexplored.

The holoenzyme of PKA is a tetrameric protein complex composed of a dimer of regulatory subunits and two monomeric catalytic subunits that each interact with one of the regulatory subunits at their C-terminal ends. Depending on the subtypes of regulatory subunits contained in the holoenzyme, PKA can be divided into type I (contains RIα or RIβ) and type II (contains RIIα or RIIβ) that utilize one of the catalytic subunits (Cα, Cβ, or Cγ) to phosphorylate proteins. PKA is activated by the rise of intracellular concentration of cAMP that binds regulatory subunits, leading to protein conformational changes and the release of catalytic subunits that are usually maintained in an in-active state in the holoenzyme. Intensive research in the past decades has established the basic molecular mechanisms underlying the versatile roles of PKA through the phosphorylation of a wide array of substrates, including transcription factors, signaling molecules, and cell surface proteins [18]. However, the specificity and efficacy of PKA can be achieved through multiple mechanisms that influence the precise timing and subcellular localizations of PKA activities. For example, PKA subunits are expressed in a cell-type specific manner. During spermatogenesis in rats, PKA-RIα was found to express earlier than RIIα (with the latter eventually becoming dominant in mature sperm), whereas Cα was found to be constitutively expressed [19,20]. Genetic studies in mice have shown that PKA subunits possess complementary roles in times of stress. For example, the lack of PKA RIIα due to gene deletion could induce the expression of RIα in order to maintain the control of the catalytic subunit [21]. It appears that PKA RIα usually plays a dominant regulatory role for PKA in cells [22]. Studies in the past have also uncovered a class of adaptor proteins (namely protein-kinase-A-anchoring proteins or AKAPs) that interact with PKA regulatory subunits and confine the PKA to sub-cellular domains [18,23]. Both type I and type II PKA regulatory subunits can bind specific AKAPs, whereas both bind dual-specificity AKAPs. In mouse testes, several AKAPs have been revealed via either biochemical or genetic studies, including AKAP3 and AKAP4 that are localized in fibrous sheath and AKAP84 that is associated with mitochondria [24,25,26,27]. Uncovering functions of AKAPs will facilitate our understanding of PKA signaling during spermatogenesis.

During spermiogenesis, type I PKA is expressed in round and elongating spermatids but diminished when mature sperm are formed, whereas type II PKA is expressed throughout the post-meiotic stage and persists into matured sperm [19,28]. These expression patterns of PKAs suggest that different sub-types of PKA play varied functional roles during spermatogenesis. In support of this notion, we found that type I PKA co-sediments with polyribosome fractions of mouse testes lysates on sucrose gradient and is involved in the regulation of protein synthesis during spermiogenesis [28], whereas type II PKA that is maintained in mature sperm may play a dominating role during the regulation of sperm motility. Consistently, AKAP3 and AKAP4, two PKA-anchoring proteins that are specifically expressed in mouse testes, have been found to be indispensable for the development and function of sperm. Gene targeting in mice showed that the absence of AKAP3 or AKAP4 disrupted the morphogenesis of sperm, giving rise to malformed sperm with greatly reduced motility, phenotypes that are often found in human astheno-teratozoospermia patients [7,29]. Specifically, *Akap3* deletion was found to cause a reduction of PKA RIα and Cα during testicular development, reminiscent of PKA RIα haploinsufficiency mutation in mice [7]. These mice carry sperm with abnormal morphology, which is similar to the phenotype of Carney complex syndrome in humans [17,30,31].

In order to investigate the molecular mechanisms that govern PKA signaling during mouse spermiogenesis, in the present study, we applied a yeast two-hybrid screen to identify potential PKA-RIα interacting proteins using a cDNA library constructed from mouse post-meiotic spermatogenic cells, mainly constituting the round and elongating spermatids. The result showed that PKA-RIα could potentially interact with a wide array of proteins that are involved in various aspects of cellular functions, including protein synthesis, RNA modification, and cytoskeletal remodeling. Many of them are testis-specific or testis-enriched proteins of which molecular functions have not been fully explored. These results give us a glimpse into the inner work of the cellular morphogenesis of spermatids and may facilitate the future exploration of mechanisms that underlie the functions of type I PKA during protein synthesis and sperm morphogenesis.

## 2. Materials and Methods

### 2.1. Animal Handling and Usage

Adult male C57/BL6 mice were used for the extraction of testes and the isolation of spermatogenic cells. Mice were first anesthetized with CO_2_ and then sacrificed by cervical dislocation. All animal husbandry and handling were carried out according to the guidelines of IACUC at Guangzhou Institutes of Biomedicine and Health, Chinses Academy of Sciences (Permit No. 2020-163).

### 2.2. Velocity Sedimentation of Mouse Spermatogenic Cells

Spermatogenic cells at different developmental stages were isolated from the testicular cells of adult mice using a velocity sedimentation method as described previously [7]. Briefly, the tunica albuginea of testes were removed, and seminiferous tubules were cut into small pieces in pre-warmed DMEM Basic media (Thermo Scientific, C11995500BT) containing 2 mg/mL of Collagenase IV (Worthington, LS004188) and 5 μg/mL of DNase I (Worthington, S0M12199) and incubated in a 37 °C humidified incubator with 5% CO_2_ for 15 min. Spermatogenic cells were dissociated by occasionally pipetting the minced seminiferous tubules up and down. Trypsin (Worthington, LS02119) was then added to a final concentration of 0.5 mg/mL, and incubation was continued for another 20 min with occasional pipetting. Dissociated spermatogenic cells were collected into a 15 mL Corning tube and spanned down at 800 rpm at room temperature (RT) for 10 min with a table centrifuge (Zonkia, SC3610, Hefei, China). Cell pellets were resuspended with 10 mL of 1X ice-cold PBS (Phosphate-buffered saline, pH 7.4) after removing the supernatant and precipitated again at 1500 rpm and RT for 10 min. Precipitated cells were resuspended in 10 mL of 1X PBS and laid onto a prepared 2–4% bovine serum albumin (BSA) gradient and sedimented for 2.5 h (hour) at RT. Fractionated cells were manually collected into 5 mL fractions in 15 mL Corning tubes. Cell types were examined using a light microscope (EVOS_fl_, Advanced Microscopy Group) according to their morphologies. Round and elongating spermatids were pooled together for total RNA extraction and cDNA preparation. From one adult male mouse, about 5 × 10^6^ round and elongating spermatids could be isolated.

### 2.3. Total RNA Extraction

Total RNA was extracted from isolated round and elongating spermatids using a RNA Simple Total kit (Tiangen, DP419, Beijing, China). Briefly, ~1 × 10^7^ cells were lysed in 1 mL of RZ buffer provided in the kit for 5 min at RT. Then, 0.2 mL of chloroform was then added and mixed by vortexing. After centrifuging for 10 min at 12,000 rpm and 4 °C, the upper layer clear phase was transferred into a fresh Eppendorf tube and mixed with a half volume of ice-cold ethanol. The mixture was added to the CR3 column provided in the kit. After washing the column with RD and RW buffers, the bound RNA was eluted with 40 mL of RNase-free H_2_O. The concentration of the total RNA was measured using spectrophotometry, and RNA bands were examined using agarose gel electrophoresis. Total RNA was stored at −80 °C.

### 2.4. Construction of cDNA Library from Mouse Round and Elongating Spermatids

A cDNA library that is suitable for the yeast two-hybridization screening of PKA-RIα interacting partners during mouse spermiogenesis was constructed using Make Your Own “Mate and Plate^TM^” kit (CloneTech, Cat.630490, Mountain View, CA, USA). Briefly, first-strand cDNAs were reverse-transcribed from the spermatids’ total RNA using SMART MMLV Reverse Transcriptase and primers containing oligo-dT30 and a short homologous sequence to the cloning site of pGADT7-Rec at the 3′-end. 1–3 nucleotide variables (VN) were added in front of oligo-dT30 in order to ensure that the amplified cDNAs would contain in-frame coding sequences of proteins (CDSIII). The first-strand cDNA was further amplified once a 5′-end primer containing a short homologous sequence was placed at the cloning site of pGADT7-Rec at the 5′-end (SMARTIII Oligo contained in the kit). The double-strand cDNAs were then amplified by PCR using primers (5′-PCR and 3′-PCR primers) containing short homologous sequences at both 5′- and 3′-ends, respectively, for the integration of cDNAs into pGADT7-Rec. From ~1 × 10^7^ spermatids, about 8 μg of cDNAs were generated. They were then used to construct a cDNA prey library in pGADT7-Rec using the homologous recombination method. Next, 3 μg of pGADT7-Rec were linearized with SmaI and co-transformed with cDNAs (7.5 μg) into 600 μL of yeast Y187 competent cells using PEG/LiAc (45 min, 30 °C, shaking) and 42 °C heat shock (20 min). The homologous recombination taking place in the cells allowed cDNAs to be inserted at the SmaI site of pGADT7-Rec. Transformed cells were first grown in YPD Plus media at 30 °C for 90 min by shaking at 250 rpm, and then they were plated onto fifty 15 cm pre-warmed SD-Leu plates. After 3–4 days, yeast colonies (average of 1–2 mm in diameter) were collected using freeze media (YPDA/kanamycin^+^ medium and 75% glycerol mixed at a 2:1 ratio at 5 mL/per plate), concentrated into 45 mL total, and stored in aliquot at −80 °C. The efficiency of transformation (colony-forming unit, CFU/μg DNA) and the titer of the cDNA library obtained (CFU/mL) were determined using the series dilution method; they were calculated to be ~2.7 × 10^5^/μg and ~6 × 10^7^/mL, respectively. The size of the inserts contained in the cDNA library were examined using PCR with primers flanking the cloning sites of pGADT7-Rec (5′- and 3′-AD LD insert primers) and agarose gel electrophoresis. DNA sequences of some of the amplified inserts were also determined with direct DNA sequencing. All used primers are listed in Supplementary Table S1.

### 2.5. Yeast Two-Hybrid Screen

Yeast culture and transformation were performed according to standard procedures. Y2HGold (MATa, Zoman Biotechnology, ZK284, Beijing, China) was used for PKA-RIα (NM_021880) bait plasmid transformation. Y187 yeast cells (MATα, CloneTech, Cat.630457) were used for prey plasmid transformation. Competent cells were prepared using 1 M LiAc (Coolaber, YT0002, Beijing, China) and the heat shock method. The culture media and plates used for yeast cells included: YPDA medium (Coolaber, PM2011), YPD plus medium (Coolaber, YT0004), YPDA agar medium (Coolaber, PM2021), minimal SD base (Coolaber, PM2030), -Leu DO supplement (CloneTech, Cat.630414), -Trp DO supplement (CloneTech, 630413), -Leu/-Trp DO supplement (CloneTech, Cat.630417), -Ade/-His/-Leu/-Trp DO supplement (CloneTech, 630428), X-α-Gal (Yeasen, 10903ES72), and Aureobasidin A (CloneTech, 630466). Then, 50 μg/mL of kanamycin were added into yeast culture media to prevent pathogen infections.

The bait plasmid expressing PKA-RIα fused with the GAL4 DNA-binding domain (GAL4-DBD) was cloned into pGBKT7 (CloneTech, Cat.630489). Primers containing NdeI and BamHI restrictive enzyme digestion sites were used to amplify the full-length cDNA of *Prka1a* and ligated into pGBKT7 following enzyme digestion. The bait plasmid contained a coding sequence for the MYC tag at the 5′-end upstream of the inserted *Prka1a* cDNA. Ligated pGBKT7-RIα was transformed into Y2HGold competent yeast cells and grew on SD-Trp plate. Yeast two-hybrid screen was performed by crossing Y187 and Y2HGold yeast strains that contained the prey cDNA library pGADT7-Rec-Library and the bait pGBKT7-RIα, respectively. Briefly, 50 mL of Y2HGold transformed with pGBKT7-RIα were freshly grown overnight (OD_600_ > 0.8). After centrifugation, cells were resuspended in 5 mL of media and mixed with 1.5 mL of Y187 containing the previously prepared pGADT7-Rec-Library. The mixture was diluted with 45 mL of YPDA/Kan^+^ media and grown at 30 °C for 24 h by shaking slowly (40 rpm). The yeast cells were then precipitated and re-dissolved into 10 mL of YPDA/Kan^+^ media and plated onto fifty 15 cm QDO/X/A plates. After 5–7 days, blue colonies that appeared were picked and inoculated onto fresh QDO/X/A plates. The positive colonies from this primary screen were then sub- individually cultured for further verifications. The primary screen resulted in total of ~50 positive clones.

### 2.6. cDNA Cloning and DNA Sequencing

Plasmids from selected blue colonies were first isolated from yeast using a Yeast Plasmid Extraction kit (Solarbio Life Science, D1160, Beijing, China). They were sequenced using the 5′-end primer. Corresponding genes were compared to the NCBI database (Ref-Seq) using the obtained DNA sequences. In order to verify the interactions between RIα and the potential proteins encoded by these genes, inserts of pGADT7-Rec were amplified using primers flanking the multiple cloning sites and ligated into pGADT7-AD using the homologous recombination method as described previously. For some genes, full-length cDNAs were also sub-cloned from an initial cDNA library using gene-specific primers containing short homologous sequences to pGADT7-AD. They were then sub-cloned into pGADT7-AD using the homologous recombination method. In total, 15 full-length cDNAs and 25 partial cDNAs were cloned into pGADT7-AD and transformed into Y187. They were used as the prey and individually hybridized with Y2HGold containing the bait pGBKT7-RIα. pGADT7-Cα and pGADT7-T antigens were used as the positive and negative controls, respectively. Of ~50 primary positive colonies, 27 were verified in the secondary screen as the potential PKA-RIα interacting partners. Primers used in the PCR and DNA sequencing reactions are listed in Table S1.

### 2.7. Database Search and Sequence Comparison

cDNAs isolated from yeast or cloned using RT-PCR from RNA samples extracted from mouse testes were sequenced. Sequences were compared using the Blast-n search on the NCBI, and corresponding genes were identified. Genes whose partial or full-length cDNAs clones were verified during the secondary yeast two-hybrid screen were grouped and analyzed using DAVID (https://david.ncifcrf.gov/home.jsp, accessed on 3 December 2020) and String (https://string-db.org, accessed on 3 December 2020) for their enriched functional preferences and putative interaction networks, respectively.

### 2.8. Western Blotting

Tissue lysates were prepared using testes from C57BL/6 mice at various ages. Briefly, testes were first extracted from mice and removed of tunica albuginea. They were then briefly homogenized on ice using a glass homogenizer in a lysis buffer (150 mM NaCl, 50 mM Tris, pH 7.2) containing 1% Triton X-100, 1X Protease Inhibitor Cocktail (Roche, 04693132001, Schaffhausen, Switzerland). The tissue homogenates were further incubated by rotating slowly at 4 °C for 2 h. Undissolved materials were discarded after centrifugation at 12,000 rpm and 4 °C for 15 min using a refrigerated tabletop centrifuge (Eppendorf, 5427R, Enfield, CT, USA). The supernatants were transferred into fresh Eppendorf tubes, and protein concentrations were measured by spectrometry. Western blotting was done according to standard procedures after proteins were separated using SDS-PAGE. The primary antibodies used were: mouse monoclonal anti-MENA (Santa Cruz Tech., sc135988, Dallas, TX, USA, 1:2000), and rabbit polyclonal anti-α-TUBULIN (Proteintech, 11224-I-AP, Rosemont, IL, USA, 1:5000). The secondary antibodies used were: goat-anti-mouse-HRP (Multisciences, GAM007, Hangzhou, China) and goat-anti-rabbit-HRP conjugates (Multisciences, GAR007, Hangzhou, China).

## 3. Results and Discussion

### 3.1. Construction of cDNA Library from Round and Elongating Mouse Spermatids

**Figure 1:**
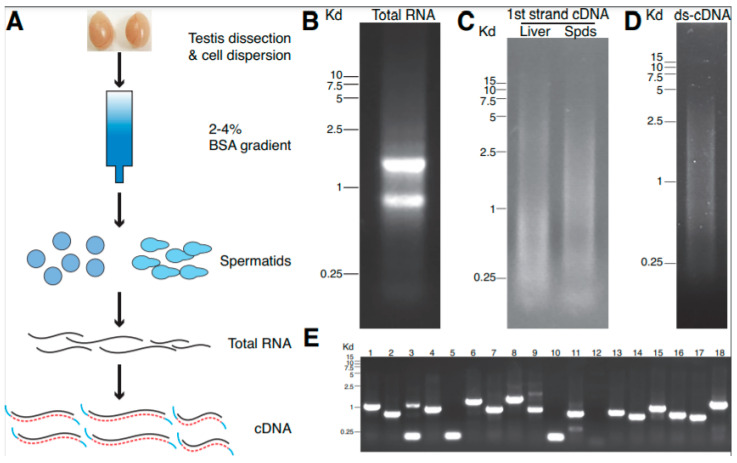
Preparation of cDNA library from mouse round and elongating spermatids. (**A**) Schematics of preparation of cDNA library from mouse haploid spermatogenic cells. (**B**) Total RNA extracted from isolated haploid spermatids. A representative image of agarose gel electrophoresis of total RNA extracted is shown. (**C**) The first-strand cDNA. First-strand cDNA was generated using the oligo-dT primer. An image of first-strand cDNA with a size of around 0.2–2 kb is shown, with the cDNA of liver RNA used as a comparison. (**D**) Double-strand cDNA from haploid spermatids. Following the amplification of double-strand cDNAs with random primer sets, the length of the obtained cDNA was around 0.5–2.5 kb. (**E**) PCR of cDNAs. Using primers flanking the cloning sites of pGADT7-Rec, clones of cDNA with various lengths were amplified and represent a diversity of genes in the library.

PKA plays diverse roles during mouse spermatogenesis. Depending on the spatial-temporal expression of various PKA subunits, different sub-types of PKA holoenzymes form in spermatogenic cells at different developmental stages [19], suggesting that different sub-types of PKA may have different functions in spermatogenic cells. In addition, AKAPs regulate sub-cellular localizations of PKA via their interactions with PKA regulatory subunits, adding another layer of control for the specificity and efficacy of PKA activities. How PKA elicits its roles using different subtypes and how subtypes of PKA localize and modify various substrates during spermatogenesis remain to be fully explored. Since PKA regulatory subunits often mediate interactions with AKAPs and substrates, identifying PKA-RIα interacting proteins would help to understand how type I PKA regulates protein synthesis during the late stage of spermatogenesis. In this regard, the yeast two-hybrid screen is a common method for un-biased protein–protein interaction discovery. However, a suitable cDNA library that contains cDNAs encoding all proteins that present in cells of interest is critical to identify target proteins. Since a whole-testis cDNA library may under-represent genes that are enriched at post-meiotic stages, we first isolated RNAs from purified haploid spermatids and constructed a post-meiotic cDNA library. As shown in Figure 1A, round and elongating spermatids could be separated from the rest of spermatogenic cells using a 2–4% BSA gradient. The purity of isolated round and elongating spermatids using this method was routinely >95% [28], thus allowing for purification of haploid spermatogenic cells and the enrichment of RNAs encoding for proteins that mainly present during spermiogenesis. Following RNA extraction and RT-PCR, post-meiotic cDNAs were cloned into a yeast vector pGADT7-Rec in order to construct the cDNA library. The size of cDNAs that were contained in the library was found to be mainly ~1 kb, ranging from 0.2 to 2.5 kb (Figure 1B–E). The copy number of cDNAs cloned in the library and the colony-forming unit (CFU) were estimated to be ~1 × 10^7^ and ~2 × 10^6^, respectively, ensuring the ample coverage of protein coding genes in haploid spermatids and the quality of the library suitable for yeast two-hybrid screen.

### 3.2. Yeast Two-Hybrid Screen of PKA-RIα Interacting Proteins

**Figure 2:**
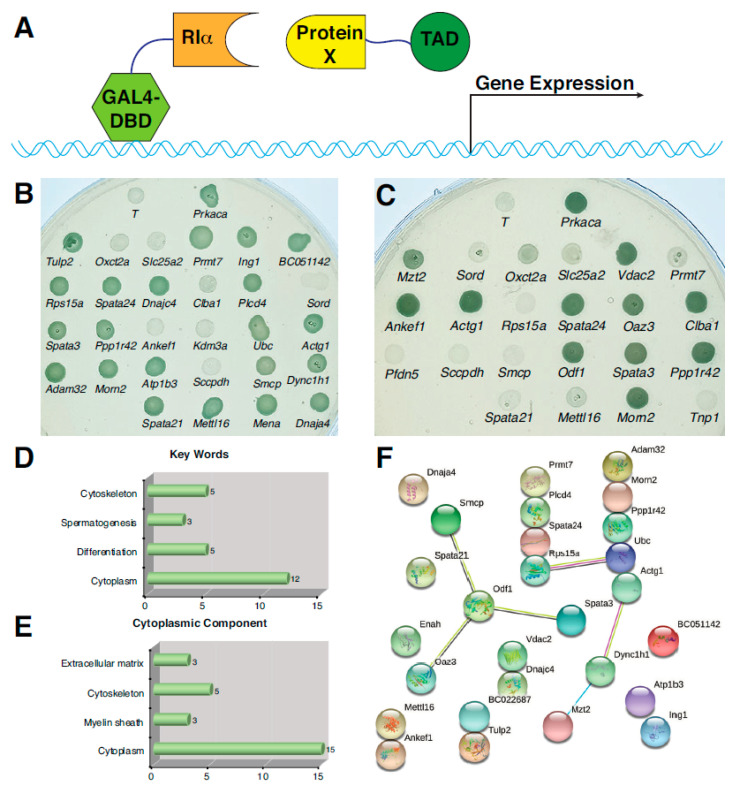
Yeast two-hybrid screen of PKA-RIα interacting proteins. (**A**) Schematic of yeast two-hybrid screen. (**B**) Secondary Y2H screen of partial cDNA clones. Green colonies are positive clones that showed interactions with bait *Prkar1a* on the quadruple drop-out QDO/X/A plates. *Prkaca* and *T* were used as positive and negative controls, respectively. (**C**) Secondary Y2H screen of full-length cDNA clones. Green colonies are positive clones that showed interaction with bait *Prkar1a* on the quadruple drop-out QDO/X/A plates. *Prkaca* and *T* were used as positive and negative controls, respectively. (**D**,**E**) GO analysis of proteins that showed positive interactions with PKA-RIα during the Y2H screen. (**F**) String analysis of putative PKA-RIα interacting proteins.

Next, we sub-cloned cDNA encoding PKA-RIα into pGBKT7 as the “bait” and conducted the yeast two-hybrid screen using a library carrying post-meiotic cDNAs in pGADT7-Rec (Figure 2A). The initial screens generated ~50 positive colonies that were able to grow on a quadruple drop-out media for yeast. Each of the colonies were then isolated and cDNAs were amplified for sequencing. A comparison of DNA sequences suggested that these putative clones encode 28 partial and 22 full-length cDNAs encoding various proteins. To verify the primary screening results, a second-round screen was carried out using individual cDNA clones. All putative pGADT7-Rec-Preys were individually co-transformed into yeast together with pGBKT7-RIα and placed in the quadruple drop-out media. Positive clones showed typical blue colonies that indicated the expression of selection markers that were under the control of the GAL4 promoter (Figure 2B–C). cDNAs encoding PKA-Cα and large T antigen were cloned into pGADT7-Rec and used as positive and negative controls, respectively. A total of 27 cDNAs were identified as positive clones in the secondary screen, including 21 partial cDNAs (Figure 2B) and 11 full-length cDNAs (Figure 2C). A database search suggested that 20 of them are either testis-specific or testis-enriched (Table S2), with some of them shown to specifically express during spermiogenesis [32,33]. Although genetic studies have shown that several of these cDNAs are important for the development of mouse sperm, including ADAM32 (A Disintegrin and Metalloproteinase domain-containing Protein 32), OAZ3 (Ornithine Decarboxylase Antizyme 3), ODF1 (Outer Dense Fiber Protein 1), SMCP (Sperm Mitochondria-associated Cysteine-rich Protein), SAPTA21 (Spermatogenesis-Associated 21), and SPATA24 (Spermatogenesis-Associated 24) [33,34,35], the functional roles for the majority of these proteins during spermatogenesis remain undetermined. The potential PKA-Riα interacting proteins that were identified were also shown to contain numerous proteins that are directly associated with protein translation machinery and protein quality control systems, such as RPS15a (Ribosomal Protein S15a), DNAJA4 (DnaJ Heat Shock Protein Family, Hsp40, Member A4), DNAJC4 (DnaJ Heat Shock Protein Family, Hsp40, Member C4), and UBC (Ubiquitin C). RPS15a belongs to the uRPS8 family, a component of a small ribosomal subunit that has been shown to positively regulate cell proliferation [36]. Both DNAJA4 and DNAJC4 are testis-enriched chaperonin proteins that facilitate the folding of nascent polypeptides, whereas UBC is a ubiquitin C family protein that controls the ubiquitination and degradation of mis-folded proteins.

### 3.3. Putative PKA-RIα Interacting Proteins during Mouse Spermatogenesis

Gene ontology analysis suggested that the 27 proteins verified during the yeast two-hybrid screen are distributed in several cytoplasmic locations and involved in a defined set of biological functions (Figure 2D–E). One of the hallmarks of spermiogenesis is the re-arrangement and re-modeling of intracellular organelles in developing spermatids. At the early stage, the fusion of Golgi cisternae and secretory vesicles ensures the formation of acrosomes. Mitochondria also undergo fusion and growth in order to form the mitochondrial sheath encircling the mid-piece of sperm tail. How the organelle re-modeling and protein synthesis are coupled during sperm morphogenesis is not clear. Interestingly, several proteins identified in the yeast two-hybrid screen are associated with different cellular organelles, such as the Golgi apparatus, mitochondria, and nucleus, including ANKEF1 (Ankyrin Repeat and EF-hand Domain Containing 1), CLBA1 (Clathrin-Binding Box of Aftiphilin Containing 1), MORN2 (MORN Repeat Containing 2), and SMCP. SMCP closely attaches to mitochondrial capsules. The lack of *Smcp* due to gene deletion in mice causes defects in sperm motility that lead to male sterility [34]. It was shown that SMCP translation during spermiogenesis is tightly controlled by its 5′- and 3′-UTRs [37]. Consistent with this notion, SMCP shows potential interactions with ODF1 and OAZ3. While the former has been shown to regulate the outer dense fiber and mitochondria sheath of sperm [35], the functional roles of the latter, as well as the other proteins, during spermiogenesis are largely unclear (see below) [38]. Other vesicle- and mitochondria-associated proteins include ATP1B3 (Na^+^/K^+^ ATPase β 3) and VDAC2 (Voltage Dependent Anion Channel 2).

Several enzymes were also found among the PKA-Riα-binding proteins, including METTL16 (Methyltransferase Like 16) and OAZ3. METTL16 post-transcriptionally modifies RNA molecules via the methylation of N6-adenosine of mRNAs and U6 snRNAs [39]. OAZ3 is the testis-specific isoform of OAZ that inhibits the activity of ornithine decarboxylase during polyamine biosynthesis and modulates protein phosphatase activity [38]. Other identified enzymes include PRMT7 (Protein Arginine N-Methyltransferase 7), PLCD4 (Phospholipase C, Delta 4), and PPP1R42 (Protein Phosphatase 1, Regulatory Subunit 42) [40]. Although it is not clear whether these enzymes are involved in protein synthesis or have other functional roles, it will be of interest to understand their relationships with PKA during spermiogenesis.

A multi-functional PKA ensures that its binding partners can be used for various purposes. This could be seen by the sub-cellular localizations of the identified proteins, ranging from cytoskeleton to secretory vesicles to mitochondria. Could they be new AKAPs in spermatogenic cells and help to modulate the localized activity of PKA? Although biochemical analyses have shown that type II PKA pre-dominates the PKA composition in mature sperm [41], type I PKA could still be functional even with reduced quantity. The presence of sperm proteins that may be important for the regulation of sperm motility (e.g., SPATA3) suggests that this is highly probable. Three spermatogenesis-associated proteins (SPATA3, SPATA21, and SPATA24) have been found, among which SPATA3 and SPATA21 have been implicated in spermatids development [42,43]. Although some experimental data are available for the relationships among a few proteins, such as SMCP, ODF1, OAZ3, and VDAC2, protein-interaction networks for most of the identified PKA-Riα-binding partners have not been investigated (Figure 2F). Thus, an un-biased yeast two-hybrid screen using post-meiotic cDNA library unveiled multiple potential interacting partners of PKA-RIα with a wide array of functions.

### 3.4. Interaction between PKA-RIα and a Short Isoform of MENA Expressed in Mouse Spermatoids

**Figure 3:**
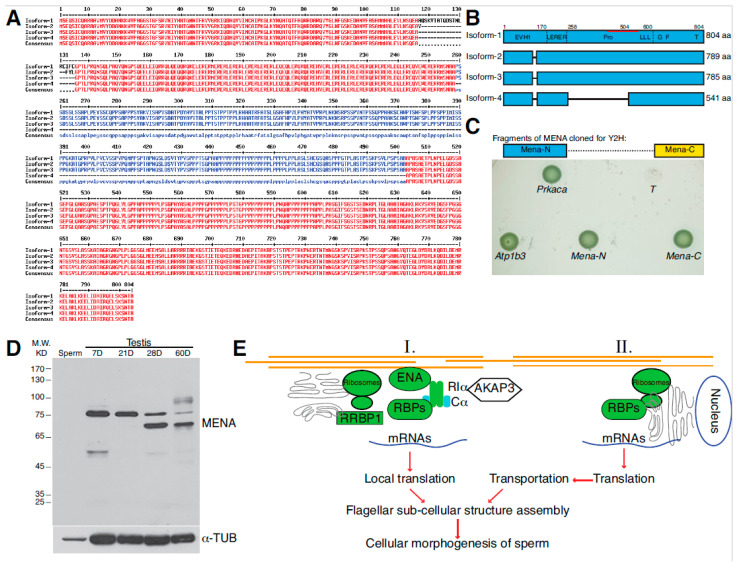
Interaction of PKA-RIα and MENA isoform expressed during mouse spermiogenesis. (**A**) Sequence alignment of MENA isoforms. The short isoform 4 lacks 244 internal amino acids compared to the other three isoforms. (**B**) Domain representation of MENA. MENA contains multiple domains that interact with various proteins. EVH1: EVH1 domain; LERER: LERER repeats; Pro: proline-rich domain; L: loading poly-proline; G: G-actin-binding site; F: F-actin-binding site; T: tetramerization site. Red line: aa415-541, a region used as the antigen for generating mouse monoclonal anti-MENA used in (**D**). (**C**) Y2H of MENA fragments. Schematics showing the fragments of MENA separated during cDNA sub-cloning (upper panel). The results show that PKA-RIα was able to interact with both the N0 and C-terminal halves of MENA (lower panel). (**D**) Western blotting of mouse testis lysates. Testes from mice at various ages were lysed and subjected to immunoblotting with anti-MENA. (**E**) A model showing protein synthesis during mouse spermiogenesis that is potentially regulated by PKA-I via interactions with MENA- and RNA-binding proteins. Proteins may be synthesized locally or near the nucleus and then transported along developing flagella to facilitate cellular morphogenesis.

Among the putative partners, sequences for two clones were initially largely mapped to 3′-UTR regions of *Atp1b3* and *Mena* (Mouse Ena/Vasp homolog). In order to ensure that these two colonies were not false-positive, cDNAs encoding ATP1B3 and MENA were separately cloned from testis RNA via RT-PCR using gene-specific primers and tested again using the yeast two-hybrid assay (Figure 3C). MENA is an actin cytoskeleton remodeling protein that is expressed in various cell types, including neurons [44]. Four isoforms of MENA have been found, among which three are long isoforms containing 804, 789, and 784 amino acids (Figure 3A). The short isoform 4 contains 541 amino acids and lacks the internal 244 amino acids encoded by exon-6 compared to isoform 1. MENA has been shown to be a modular protein that contains an N-terminal EVH1 domain that is highly homologous to ENABLED and VASP actin-binding proteins [45]. Its central region contains LERER repeats and proline-rich domain, whereas its C-terminal region contains G- and F-actin-binding domains, as well as the tetramerization domain (Figure 3B). In order to examine which isoform is expressed in mouse haploid spermatids and which region of MENA mediates the interaction with PKA-RIα, cDNAs encoding the N-terminal, central, and C-terminal regions of MENA were separately cloned y using gene-specific primers via RT-PCR. While we successfully obtained cDNA fragments for the N- and C-terminal regions (Mena-N and Mena-C, respectively), the central region matching the missing nucleotides in isoform 4 could not be amplified, suggesting that mouse haploid spermatids mainly express the short isoform of MENA. The yeast two-hybrid assay showed that both the N- and C-terminal domains of MENA interact with PKA-RIα (Figure 3C). We further analyzed the expression of MENA in mouse testes using Western blotting. Testis lysates were prepared from mice at different ages, ranging from neonates (7 dpp (days post-partum)) to adults (60 dpp). Two protein bands with molecular weights of 80 and 70 kilodalton (KD) were found with the Western blot. The shorter protein band appeared around four weeks of age when haploid spermatids entered the elongating stage and gradually dominated when mice reached more advanced ages. This result suggested that the long isoform of MENA is expressed early during spermatogenesis, whereas the shorter MENA isoform 4 is expressed when spermiogenesis initiates, consistent with the result of the yeast two-hybrid screen (Figure 3D).

In mammalian neurons, ENAH has been shown to regulate the RNA granule dynamics and facilitate the local translation of proteins in the synapse in response to stimulating signals [46]. It also binds the barbed ends of F-Actin and regulates the dynamic assembly of cortical actin filament, thus helping to modify the morphology and migration of cells [45]. Although PKA has been implicated in the same cellular pathways of protein synthesis and synaptic changes in neurons [47,48], the relationship between PKA and ENA/VASP is not clear. The interaction between PKA-RIα and MENA in developing spermatids could provide the molecular link between signal transduction and protein synthesis that occurs in either a localized or dynamic fashion (Figure 3E). In the same vein, the identified cytoskeletal proteins, including ACT1α (Cytoplasmic α-ACTIN-1), DYNC1H1 (Dynein Cytoplasmic 1 Heave Chain 1), and MZT2 (Mitotic Spindle Organizing Proteins 2), are believed to regulate cargo transport and mitotic spindle dynamics [49], which would help to modify the morphology of developing spermatids. Whether they are indeed modified by PKA and subsequently regulate protein homeostasis during spermiogenesis requires further investigation.

The elucidation of protein–protein interactions often helps to unveil the molecular mechanisms that underlie protein functions. Several studies have used the yeast two-hybrid method to investigate PKA-binding proteins using either PKA-C or PKA-R as the bait, which has helped to find new AKAPs and substrates in various types of cells [50,51,52]. However, the systemic identification of PKA-binding proteins in spermatogenic cells has not been done before despite the fact that PKA participates in the regulation of numerous aspects of spermatogenesis and sperm function. In the present study, we applied a yeast two-hybrid screen system to uncover PKA-RIα-binding partners in haploid spermatogenic cells. Although this heterologous system has the advantage of high efficiency, overexpressed exogenous proteins sometimes generate false positives. To minimize this problem, we constructed a cDNA library specifically from haploid spermatids in order to enrich proteins that express at the desired developmental time, conducted primary and secondary screens to eliminate false positives, and included both positive (PKA-Cα) and negative (large T antigen) controls for individually verified interactions. However, whether the identified proteins represent physiological partners of PKA-RIα requires further investigation. Experiments including biochemical validations of direct protein–protein interactions, co-localizations of these proteins in spermatogenic cells, and the functional roles of their interactions using mouse genetics should be carried out in the future. Nonetheless, a yeast two-hybrid screen provided us an unbiased assay to unveil protein-interaction networks encompassing a variety of cellular processes. Deciphering the relationships between these proteins and PKA and their functional roles during spermatogenesis would broaden our understanding of how PKA signaling is precisely controlled in time and space, as well as molecular mechanisms underlying the regulation of the cellular morphogenesis of the male gamete.

## Data Availability

The data presented in this study are available in Supplemental Tables S1 and S2.

## Supplementary Materials

The following are available online at https://www.mdpi.com/article/10.3390/genes12121941/s1, Table S1: List of primers used; Table S2: List of genes identified to interact with PKA-RIα.
