# Supplementary material for: Yeast Two-Hybrid Screen Identifies PKA-Riα Interacting Proteins during Mouse Spermiogenesis
Source: Genes (Basel). 2021 Nov 30;12(12):1941. doi: 10.3390/genes12121941 (PMC8700991; doi:10.3390/genes12121941)
Supplement: Supplementary file 1 [file genes-12-01941-s001.zip › genes-1447070-supplementary.pdf]

Supplementary Table-S1

Supplementary Table-S1: List of primers used.	
**Primers fo construction of cDNA library**	
**SMART III Oligo**	5’-AAGCAGTGGTATCAACGCAGAGTGGCCATTATGGCCGGG-3’
**CDSIII primer**	5’-ATTCTAGAGGCCGAGGCGGCCGACATG-d (T) 30 VN-3’
**CDSIII/6 primer**	5’-ATTCTAGAGGCCGAGGCGGCCGACATG-NNNNNN-3’
**Primers for cDNA library amplification**	
**5'-PCR Primer**	5’-TTCCACCCAAGCAGTGGTATCAACGCAGAGTGG-3’
**3'-PCR Primer**	5’-GTATCGATGCCCACCCTCTAGAGGCCGAGGCGGCCGACA-3’
**Primers for cDNA of Prkar1α (Underlined: Ned1 and BamHI sites)**	
**X144F**	5’-GGAATTCCATATGATGGCGTCTGGCAGTATGGC-3'
**X144R**	5’-CGGGATCCTCAGACGGACAGGGACACGAAGC-3'
**Primers for identification of plasmids in yeast**	
**5'-AD LD insert primer**	5'-CTATTCGATGATGAAGATACCCCACCAAACCC-3‘
**3'AD LD insert primer**	5'-GTGAACTTGCGGGGTTTTTCAGTATCTACGATT-3'
**Primers for PCR of partial cDNAs for homologous recombination in yeast**	
**5'-LD insert primer**	5'-GGAGTACCCATACGACGTACC-3'
**3'-LD insert primer**	5'-TATCTACGATTCATCTGCAGC-3'
**Primers for RT-PCR of full-length cDNAs (Underlined: homologous arms for recombination in yeast)**	
**Vdac2 (888 bp)**	
**X5F**	5'-ACGACGTACCAGATTACGCTATGGCTGAGTGCTGTGTACCGGT-3'
**X5R**	5'-TATCTACGATTCATCTGCAGTTAAGCCTCCAATTCCAAG-3'
**Mzt2 (480 bp)**	
**X12F**	5'-ACGACGTACCAGATTACGCTATGGCGGCGGCGGCGGCTGCTGCT-3'
**X12R**	5'-TATCTACGATTCATCTGCAGCTATGGGCTGCTTCGGGAGTTGCTT-3'
**Clba1 (966 bp)**	
**X14F**	5'-ACGACGTACCAGATTACGCTATGCAAGGCGGGCAGGAGGT-3'
**X14R**	5'-TATCTACGATTCATCTGCAGTTAGCAGACATCATTATTAAAGAAT-3'
**Oaz3 (733 bp)**	
**X15F**	5'-ACGACGTACCAGATTACGCTCTGCCTTGTAACAGGTCCCGC-3'
**X15R**	5'-TATCTACGATTCATCTGCAGTCACTGGCCAGGGTGGC-3'
**Spata24 (588 bp)**	
**X20F**	5'-ACGACGTACCAGATTACGCTATGGCGACGCCCCTTG-3'
**X20R**	5'-TATCTACGATTCATCTGCAGTCATTTCCGTGACCTGCCT-3'
**Actg1 (733 bp)**	
**X23F**	5'-ACGACGTACCAGATTACGCTATGTTTGAAACCTTCAATACCCCAG-3'
**X23R**	5'-TATCTACGATTCATCTGCAGCTAGAAGCATTTGCGGTGGACG-3'
**Ankef1 (2328 bp)**	
**X31F**	5'-ACGACGTACCAGATTACGCTATGGCTTTGGCAGACAAGAGACT-3'
**X31R**	5'-TATCTACGATTCATCTGCAGTTAGTTCTTGAGTGTTGCCTCCAGT-3'
**Ppp1r42 (1074 bp)**	
**X34F**	5'-ACGACGTACCAGATTACGCTATGGTTCGACTGACGGTGGAT-3'
**X34R**	5'-TATCTACGATTCATCTGCAGTTACTCCTTTTTCTCAGAC-3'
**Spata3 (513 bp)**	
**X35F**	5'-ACGACGTACCAGATTACGCTATGAAGAAGGTCAAAAAGAAAAAGT-3'
**X35R**	5'-TATCTACGATTCATCTGCAGCTACACCCAAGGCTTTTTAGC-3'
**Odf1 (747 bp)**	
**X38F**	5'-ACGACGTACCAGATTACGCTATGGCCGCACTGAGTTGTCT-3'
**X38R**	5'-TATCTACGATTCATCTGCAGTTATAAGATCATCTTCCTACAG-3'
**Morn2 (240 bp)**	
**X51F**	5'-ACGACGTACCAGATTACGCTATGAATGGCTTTGGAAGACTTG-3'
**X51R**	5'-TATCTACGATTCATCTGCAGCTACATGTAGAGCTTTAGTTTCAGG-3'
**Atp1b3 (843 bp)**	
**X46F**	5'-ACGACGTACCAGATTACGCTACGAAGACTGAGAAAAAATCCT-3'
**X46R**	5'-TATCTACGATTAATCTGCAGGGCTCGTGCTGTGACTTTGAAC-3'
**Primers for RT-PCR of partial cDNAs encoding N- and C-terminal MENA fragments**	
**Mena-1-F**	5'-ACGACGTACCAGATTACGCTAGTGAACAGAGTATCTGTCAGGC-3'
**Mena-1-R**	5'-TATCTACGATTCATCTGCAGTCTGCGCTCTCGCTCCCACTC-3'
**Mena-2-F**	5'-ACGACGTACCAGATTACGCTGCATCTGGAATTTTCTCTGGATC-3'
**Mena-2-R**	5'-TATCTACGATACATCTGCAGTGCAGTGTTCGACTTGCTCAGC-3'

Supplementary Table-S2

**Supplementary Table-S2: List of genes identified to interact with PKA-RIa.**								
**Gene Symbol**	Gene Name	NCBI Accession Number	Protein ID	Protein Length	cDNA clone in pGADT7	Polypeptide encoded	Expression (ENCODE)	Fnctional Roles
**Actg1**	Actin, gamma, cytoplasmic 1	NM_009609.3	NP_033739.1	375 aa	Full-length/Partial	aa224-375	Ubiquitous	Actin cytoskeletal component
**Adam32**	A disintegrin and metallopeptidase domain 32	NM_153397.2	NP_700446.2	754 aa	Partial	aa644-754	Testis-specific	Sperm development and fertilization
**Ankef1**	Ankyrin repeat and EF-hand domain containing 1	NM_175667.4	NP_783598.1	775 aa	Full-length	aa1-754	Testis-specific	Enriched in ciliated tissue and cells, testis
**Atp1b3**	ATPase, Na+/K+ transporting, beta 3 polypeptide	NM_007502.5	NP_031528.1	278 aa	Full-length	aa1-700	Testis-enriched	Nociceptive sensitivty
**BC051142**	Testis-expressed basic protein 1 isoform 1	NM_001001177.2	NP_001001177	393 aa	Partial	aa223-363	Testis-specific	N/A
**Clba1**	Clathrin binding box of aftiphilin containing 1	NM_145450.3	NP_663425.2	321 aa	Full-length	aa1-321	Testis-enriched	N/A
**Dnaja4**	DnaJ heat shock protein family (Hsp40) member A4	NM_001357875.1	NP_001344804.1	462 aa	Partial	aa199-426	Testis-enriched	Cholesterol synthesis
**Dnajc4**	DnaJ heat shock protein family (Hsp40) member C4	NM_001356999.1	NP_001343928.1	261 aa	Partial	aa17-261	Testis-enriched	N/A
**Dync1h1**	Dynein cytoplasmic 1 heavy chain 1	NM_030238.2	NP_084514.2	4644 aa	Partial	aa4501-4644	Ubiquitous	Retrograde transport, dendritic morphology,
**Mena**	Mammalian ENA/VASP actin regulator (Enah) homolog, transcript variant 4	NM_001083121.2	NP_001076590	541 aa	Partial, partial	aa1-253, aa344-541	Ubiquitous	Actin cytoskeleton remodeling and cell polarity
**Ing1**	Inhibitor of growth family, member 1	NM_011919.5	NP_036049.2	279 aa	Partial	N/A	Ubiquitous	Inhibits p53-dependent transcription activation
**Mettl16**	Methyltransferase like 16	NM_026197.3	NP_080473.1	553 aa	Partial	aa468-553	Ubiquitous	Methylates N6-adenosine of mRNAs and U6 snRNAs
**Morn2**	MORN repeat containing 2	NM_001360440.1	NP_001347369.1	79 aa	Full-length	aa1-79	Testis-specific	Membrane occupation and recognition nexus repeat-containing-2
**Mzt2**	Mitotic spindle organizing protein 2	NM_001359003.1	NP_001345932.1	159 aa	Full-length	aa1-159	Testis-enriched	Gamma-tubulin ring complex associated Mozart2 family member
**Oaz3**	Ornithine decarboxylase antizyme 3	NM_016901.3	NP_058597.2	243 aa	Full-length	aa1-243	Testis-specific	Inhibits ornithine decarboxylase (ODC) sperm connecting piece development
**Odf1**	Outer dense fiber of sperm tails 1	NM_008757.3	NP_032783.2	248 aa	Full-length	aa1-248	Testis-specific	HSPB10, mitochondrial sheath and ODF organization, head-tail linkage, motility
**Plcd4**	Phospholipase C, delta 4	NM_001081456.1	NP_001074925.1	775 aa	Partial	aa597-775	Testis-specific	Hydrolyzes PIP2 to generate DAG and IP3, acrosome reaction
**Ppp1r42**	Protein phosphatase 1, regulatory subunit 42	NM_145692.2	NP_663730.1	357 aa	Full-length/Partial	aa1-357	Testis-specific	Positively regulates protein phosphatase 1 (PP1) in the testis, centrosome dynamics
**Prmt7**	Protein arginine N-methyltransferase 7	NM_145404.1	NP_663379.1	692 aa	Partial	aa598-692	Testis-enriched	catalyzes ω-monomethylarginine and symmetric-dimethylarginine formation
**Rps15a**	Ribosomal protein S15A	NM_170669.2	NP_733769.1	130 aa	Partial	aa86-130	Ubiquitous	uRPS8, component of ribosomal small subunit, positively regulate cell proliferarion
**Smcp**	Sperm mitochondria-associated cysteine-rich protein	NM_008574.4	NP_032600.3	143 aa	Partial	aa120-143	Testis-specific	Mitochondrial capsule associated, motility regulation
**Spata21**	Spermatogenesis associated 21	NM_177867.3	NP_808535.1	681 aa	Partial	aa621-681	Testis-specific	EF hand containing, may be involved in differentiation of haploid spermatids
**Spata24**	Spermatogenesis associated 24	NM_027733.5	NP_082009.3	185 aa	Full-length/Partial	aa1-155	Testis-specific	DNA binding, may be involved in cytoplasm movement and removal during spermiogenesis
**Spata3**	Spermatogenesis associated 3	NM_027300.3	NP_081576.2	193 aa	Full-length/Partial	aa1-159	Testis-specific	N/A
**Tulp2**	Tubby-like protein 2	NM_008807.3	NP_032833.2	562 aa	Partial	aa398-562	Testis-specific	C-terminal TUB domain, stress response, TF, PI binding, GPCR and Shh signaling
**Ubc**	Ubiquitin C	NM_019639.4	NP_062613.3	734 aa	Partial	aa649-734	Ubiquitous	Protein ubquitilation
**Vdac2**	Voltage-dependent anion channel 2	NM_011695.2	NP_035825.1	295 aa	Full-length	aa1-295	Testis-enriched	Outer mitochondrial membrane ion channel, has both anion and cation seletivity
